# Studies on CWL with glycerol for combustion process

**DOI:** 10.1007/s11356-018-3814-0

**Published:** 2018-11-28

**Authors:** Anita Staroń, Zygmunt Kowalski, Paweł Staroń, Marcin Banach

**Affiliations:** 1 Department of Engineering and Chemical Technology, Cracow University of Technology, 24 Warszawska St, 31-155 Cracow, Poland; 2 Mineral and Energy Economy Research Institute, Polish Academy of Sciences, 7 Wybickiego St, 31-261 Cracow, Poland

**Keywords:** CWS, Coal, Liquid fuel, Coal-water slurry, Glycerol

## Abstract

Findings of more efficient energy recovery methods are focused on composite fuels consisting of coal, water, and waste materials. The use of such slurry fuels has enormous environmental benefits due to the potential for waste utilization and low emissions of harmful oxides to the atmosphere during the combustion process. In this study, we report the effect of waste glycerol on the rheological properties of coal-water fuel (CWS). The addition of glycerol to CWS increases the viscosity (from 45.5 to 184.2 mPa·s at a shear rate of 100 rpm) and density (from 1.08 to 1.11 g/cm^3^) of these suspensions. The utility of choice of the most favorable parameters is equal to 0.85 for both CWS and CWS with added glycerol. Due to the fact that CWS is dosed to heated chamber, its modification with glycerol results in easier nebulization into the combustion chamber, lowering of the solidification temperature and increasing the calorific value of the resulting fuel. During combustion of CWS with glycerol, the amount of SO_2_ and NO_*x*_ emitted is reduced by half as compared to the emission of these gases during hard coal combustion.

## Introduction

The issue of increasing the efficiency of obtaining energy through power stations has been already discussed for years (Delitsyn and Vlasov 2010; Belošević et al. 2015; Bukhonov and Morozov 2003). The need to increase the energy efficiency comes not only from the limited resource of fossil fuels but above all from the need to reduce emissions of harmful substances, both gases (sulfur dioxide, carbon dioxide, nitrogen oxides) and particulates (dust and solid waste which are collected on landfills). The searches for methods of more efficient use of energy lead to make the formation of composite fuels based on coal.

The use of this type of fuels brings many advantages including the possibility of waste sludge and materials application, reduction of emissions of harmful gases to atmospheres, acquiring new energy sources, and implementing the principles of sustainable development.

Coal-water slurry (CWS) belongs to the group of composite fuels. Results of studies (Cheng et al. 2010; Dmitrienko et al. 2017; Khodakov et al. 2006; Park et al. 2017; Phuoc et al. 2014) confirm the increase in the amount of energy gained as a result of the use of CWS as coal substitution. Coal-water fuel consists of fine particles of coal, water, and various additives. The use of CWS is becoming more popular due to its environmental aspect. They can be a substitute for heating oil, which is ecologically very beneficial because of the reduction of CO_2_, SO_2_, and NO_*x*_ emissions during burning this fuel (Nyashina et al. 2017). The coal raw material of the slurry consists of potassium and calcium compounds which, in the combustion environment, contribute to the binding of sulfur compounds and the formation of a solid phase in the form of sulfates (IV) (Staroń et al. 2016). This reduces the emission of sulfur dioxide. The reduction of nitric oxide emissions results from the oxidation of these oxides to nitrogen dioxide at about 200 °C (Kępiński 1984) (temperature of the off-gas), which in turn leads to the formation of calcium and potassium nitrate, that are compounds soluble in water condensed from gases.

The calorific value of the coal-water fuel in terms of energy (for a slurry containing 40–70% of coal) ranges from 8.3 to 20.1 MJ/kg and depends on the amount of water and type of coal from which it was prepared (Buyantuev et al. 2014; Mukherjee and Pisupati 2015; Wei and Wang 2016). The maximum solids content in the slurry, in the case of monomodal particles, is about 65%, for multimodal more than 80%, as a result of fine particle settling between the larger particles. CWS should have good stability and low viscosity, so that the suspension can be properly dispensed into the combustion chamber. The lowest viscosity can be obtained at a ratio of larger to fine particle equal to 35:65 (Turian et al. 2002; Lee et al. 2007; Barners et al. 1989; Zhang et al. 2011; Staroń et al. 2013). To improve rheological properties of the coal-water suspension, there are used additives, dispersants, such as surfactants and electrolytes (Lee et al. 2007; Aktas and Woodburn 2000; Al-Amrousi et al. 1996; Seshadri et al. 2008; Verma et al. 2006; Naik et al. 2009; Mukherjee and Pisupati 2016; Mosa et al. 2008; Pat. US4416666 1979; Shin and Shen 2006; Wu et al. 2015; Zhang et al. 2016; Ma et al. 2013). The addition of waste glycerol which is a surfactant to CWS can improve the useable properties of the resulting mixtures and further increase their calorific value, which is beneficial for their later use as fuel. Moreover, high ignition temperature of glycerol assumes that it may be concerned as an interesting additive for other fuels (Gupta and Kumar 2015).

Waste glycerol is one of the major by-products of biofuel production. This is so called the glycerol phase containing glycerol, methanol, mono-, diacylglycerols, free fatty acids, and soaps (Tan et al. 2013). During the production of biofuels, the glycerol phase is always produced in excess of 12% relative to the obtained esters, irrespective of the type of catalyst, device, or technology.

Worldwide biodiesel production has been predicted to reach about 39 billion liters by 2024 (Kong et al. 2016). Huge amounts of unwanted waste glycerol have attracted the interest of scientists who are looking for ways to use it. Due to the significant demand for petroleum fuels, it is justified to receive and use composite fuel consisting of coal, water, and waste from the biodiesel process. This will help to reduce the consumed amount of petroleum fuel.

The studies envisage the use of technical glycerol as an additive to coal-water suspensions, which may be a substitute for conventional fuels. The novelty of the study was related to investigation of the physicochemical properties of coal-water suspensions modified with glycerol.

## Experimental part

### Materials

The coal raw material from which the slurry was made was flame coal with a low ash content and a grain size of 6–25 mm, and fine coal with a grain size of up to 20 mm. Both raw materials were characterized by similar calorific value, about 25.5 [MJ/kg] and sulfur content equal to 1.1%. They consisted of the following elements: Al, Si, P, S, K, Ca, Ti, and Fe. Table 1 shows the characteristics of coal raw materials.

**Table 1:** Characteristics of coal and fine coal

		Coal	Fine coal
Ash	[%]	7.9	9.9
Moisture		9.7	9.4
The volatiles		39.1	39.2
Coal		63.7	66.3
Nitrogen		4.9	5.1
Hydrogen		1.0	1.1

The used raw material is characterized by a high oxidation rate, the highest of which is achieved in about 400 °C. This involves over 80% loss of sample weight. This is due to carbon degassing. There is the distillation and pyrogenic decomposition of coal compounds, which results in the formation of gases and vapors that emit a semi-liquid mass of coal (Kijo-Kleczkowska 2011).

The percentage of individual groups of macerations in coal and fine coal does not decompose proportionately. The content of macerals of vitrinite group is dominant (53.4% in coal and 59% in fine coal). The lowest share of macerated groups after calculation into pure mineral matter is found in the group of liptinite (8% in coal, 9.2% in fine coal). Vitrinite, whose content prevails among the other macerals, is characterized by the presence of micropores, which results in worse water absorption. Macerals of the vitrinite group are the most fragile; the liptinite group is characterized by higher hardness, bonding properties, and considerable elasticity, which affects the mechanical properties. Due to its porous structure, liptinite contributes to the increase of viscosity of the coal-water mixture. Thus, its low share benefits from the viewpoint of the properties of the resulting coal-water slurry. Macerals of inertinite groups (32% in coal, 24.6% in fine coal) are characterized by higher hardness than the rest of the groups, which results in increased mechanical strength and therefore affects the efficiency of the coal grinding process. In addition, mineral matter was identified in coal and fine coal at a level of 7.9% and 9.9%, respectively.

In the study, there was used technical glycerol characterized by the purity min. 97.5% (Avantor Performance Materials Poland).

### Methods

**Fig. 1:**
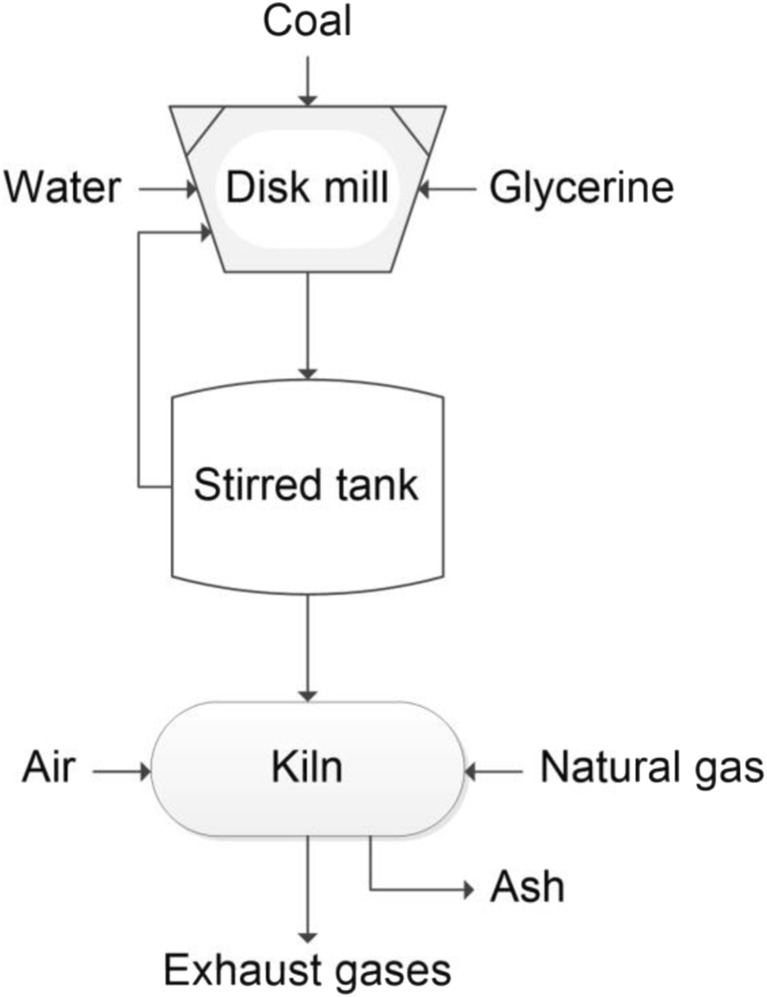
Diagram of the process of obtaining and burning coal-water slurry

The process of receiving the CWS was realized on a huge-laboratory scale in a disk mill (Staroń et al. 2016; Pat. PL409826 2014). The schematic diagram of the process for the production and combustion of coal-water slurries is shown in Fig. 1.

The installation consisted of a tank of 100 dm^3^, a pump with a capacity of 30 dm^3^/h, and a disk mill with a disk diameter of 150 mm. Mill disks specially designed for the production of coal-water suspensions were used in the process (Pat. PL397860 2012; Pat. PL224446 2016).

Coal material and water were fed to the mill. Composition of the slurry and grinding time were modified to obtain a coal-water suspension with the most advantageous utility parameters (high stability, low viscosity and density, and the smallest diameter of coal particles). In case of ternary suspensions, the third ingredient was glycerol in 10 or 20% by mass. Table 2 shows variability levels of independent variables for the two-component and ternary suspension processes.

**Table 2:** Variability levels of independent parameters for the process of coal or fine coal and water slurry and coal or fine coal, water and glycerol slurry preparation

Variable		Bottom level	Top level
CWS			
The content of coal raw materials (coal, fine coal)	[%]	50	60
The content of water		40	50
Grinding time	[h]	6	18
CWS + glyc			
The content of coal raw materials (coal, fine coal)	[%]	50	60
The content of water		30	40
The content of glycerol		10	20
Grinding time	[h]	6	18

The obtained suspensions were analyzed which made it possible to determine their usefulness properties. The equivalent diameter was determined by the direct method, based on particle observations using a Hitachi scanning microscope, model TM3000 in × 1500 magnification. The image obtained in a digital form was analyzed using NIS-Elements software for particle size analysis. The density of suspensions was determined by the pycnometric method. Rheotest RN 3.1 was used to determine a viscosity coefficient for the shear rate of 100/min and 200/min at temperature of 25 °C. Stability of the mixtures was defined as the difference in height between the surfaces of the mixtures after their preparation and the phase boundary formed by the sedimentation of coal particles after 24 h after the homogenization of the sample. As the height difference increases (*H*), the stability decreases. In order to confirm the stability of the suspensions, the zeta potential of the selected samples was also measured using Zetasizer Nano ZS firmy Malvern.

The CWS combustion process was carried out in a rotary kiln. The slurry was dispensed into the combustion chamber using compressed air through the spray nozzles of the burner. The emission measurement was made during the combustion process of the slurry in a rotary kiln, heated to 800 °C.

During the combustion of natural gas, hard coal, coal-water slurry, and coal-water slurry with glycerol, an exhaust gas analysis was performed. The amount of burned coal raw material corresponded to the average amount of coal burned in the coal-water slurry. Gas analyses were carried out using the gas analyzer Madur GA-40T Plus with built-in electrochemical sensors (O_2_, CO, NO, NO_2_, SO_2_, H_2_S, H_2_) and based on infrared absorption (CH_4_ and CO_2_). Statistical analysis of the results was conducted in Statistica v. 10 from StatSoft®. Pareto graphs were made based on the analysis of variance and they show the effects of independent variables on dependent (standardized effects). The vertical line in the graph corresponds to the assumed significance level (*α* = 0.05) and separates the statistically significant effects from the non-significant. Approximation profiles were obtained to determine the values of independent parameters that allowed obtaining most desirable estimated resulting factors. Estimated output values for each combination of input values have been converted to usability scale. The relative usability of different output values determines the usability function. The utility values of the dependent variable can vary for a value predicted from 0.0 (undesired) to 1.0 (highly desirable) (Stanisz 2007a, b).

Utility as the criterion for selecting the most advantageous parameters is defined as follows: the highest stability that will allow storing the product, the smallest particle diameter that affects stability, and the possibility of introducing CWS into the burner, the lowest density and viscosity that determine the transportability of the slurry and allow its dispensing into the furnace using nozzles. For such (beneficial) parameters, the usability function assumes a value of 1.

## Results and discussion

### Physicochemical properties of CWS

The coal-water and coal material-glycerol slurry characteristics are listed in Table 3. The lack of data on viscosity coefficients in some cases is due to the fact that the consistency dense of these suspensions is too large.

**Table 3:** Process parameters and physicochemical properties of coal-water-glycerol suspensions

N	Raw material	Glycerol [%]	Water [%]	Grinding time [h]	Stability [mm]	Density [g/cm^3^]	Viscosity 100/min [mPa·s]	Viscosity 200/min [mPa·s]	Equivalent diameter [μm]
1	Fine coal	0	50	6	6.0	1.17	–	–	7.7
2	Fine coal	0	50	12	0.0	1.16	419.0	250.0	8.5
3	Fine coal	0	50	18	0.0	1.21	468.0	297.0	12.7
4	Coal	0	40	6	0.0	1.13	–	–	6.0
5	Coal	0	50	6	4.0	1.08	–	–	8.5
6	Coal	0	40	12	0.0	1.10	489.0	332.0	6.4
7	Coal	0	50	12	3.0	1.08	–	65.8	6.6
8	Coal	0	40	18	0.0	1.12	1530.0	1080.0	4.0
9	Coal	0	50	18	0.0	1.06	432.0	265.0	4.5
10	Coal	0	40	6	0.0	1.20	–	–	5.4
11	Fine coal	10	40	6	3.0	1.11	–	–	6.0
12	Fine coal	20	30	6	1.0	1.16	–	–	5.5
13	Fine coal	10	40	12	0.0	1.08	684.0	410.0	4.8
14	Fine coal	20	30	12	0.0	1.14	1050.0	676.0	6.4
15	Fine coal	10	40	18	1.0	1.13	–	–	5.1
16	Fine coal	20	30	18	0.0	1.12	2310.0	1650.0	6.0
17	Coal	10	50	6	0.0	1.18	–	–	3.8
18	Coal	20	40	6	0.0	1.19	–	–	7.0
19	Coal	10	40	6	2.0	1.13	–	–	5.8
20	Coal	20	30	6	2.0	1.14	–	–	7.0
21	Coal	10	50	12	0.0	1.19	678.0	476.0	5.1
22	Coal	20	40	12	0.0	1.12	1610.0	1210.0	4.5
23	Coal	10	40	12	5.0	1.08	–	–	9.9
24	Coal	20	30	12	2.0	1.13	223.0	164.0	5.3
25	Coal	10	50	18	0.0	1.12	1870.0	1480.0	5.6
26	Coal	20	40	18	0.0	1.16	1760.0	1400.0	6.4
27	Coal	10	40	18	0.0	1.11	–	–	4.4
28	Coal	20	30	18	2.0	1.17	485.0	328.0	5.8

**Fig. 2:**
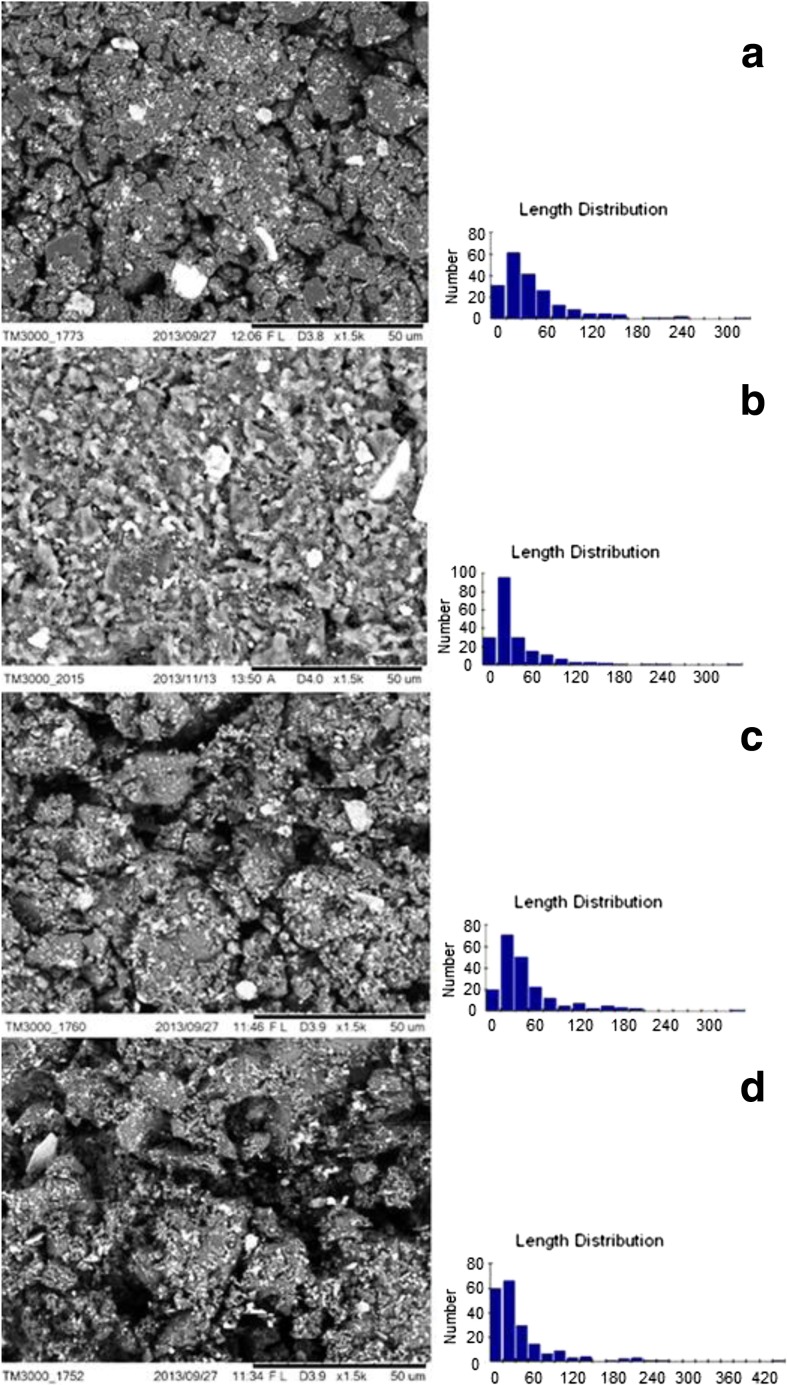
SEM micrograph and particle size distribution in the slurry of **a** coal-water-glycerol slurry after 6-h milling (sample no. 20), **b** fine coal-water-glycerol suspension after 6-h milling (sample no. 12), **c** coal-water slurry after 6-h milling (sample no. 5), and **d** coal-water-glycerol slurry after 12-h milling (sample no. 24)

Figure 2 shows the size and shape of the coal particles in the selected slurries. Coal particles in these slurries are characterized by high fragmentation, irregular shape, and considerable variation in size. It is observed in coal-water-glycerol slurry after 6 h of grinding strong packing of coal particles (Fig. 2a and b), which results in high stability. This suspension is dominated by particles of 15–30 μm in diameter. Figure 2c shows, for comparison, the coal-water slurry particles after 6 h. Relatively, high particle stability is observed for suspensions in which the diameter of particles is around 30 μm, which promotes the formation of agglomerates. Comparing the slurries obtained from the same raw material at the same grinding time, the slurries having their glycerol content (Fig. 2a and c) are more stable. In the case of a 12-h grinding of coal-water-glycerol slurry, a strong packing of particulate particles was observed, which positively influences the stability of the resulting slurry (Fig. 2d).

In addition, suspensions with high stability (slurry no. 8, 25, and 26), obtained by 18-h grinding of coal, differing in water and glycerol content, were selected and the zeta potential was determined. Zeta potential values for suspensions no. 8, 25, and 26 are − 30.1 mV, − 30.7 mV, and − 24.9 mV respectively. Absolute zeta potential values above 30 mV provide good stability. About 20 mV, they provide only short-term stability (Honary and Foruhe 2013).

**Fig. 3:**
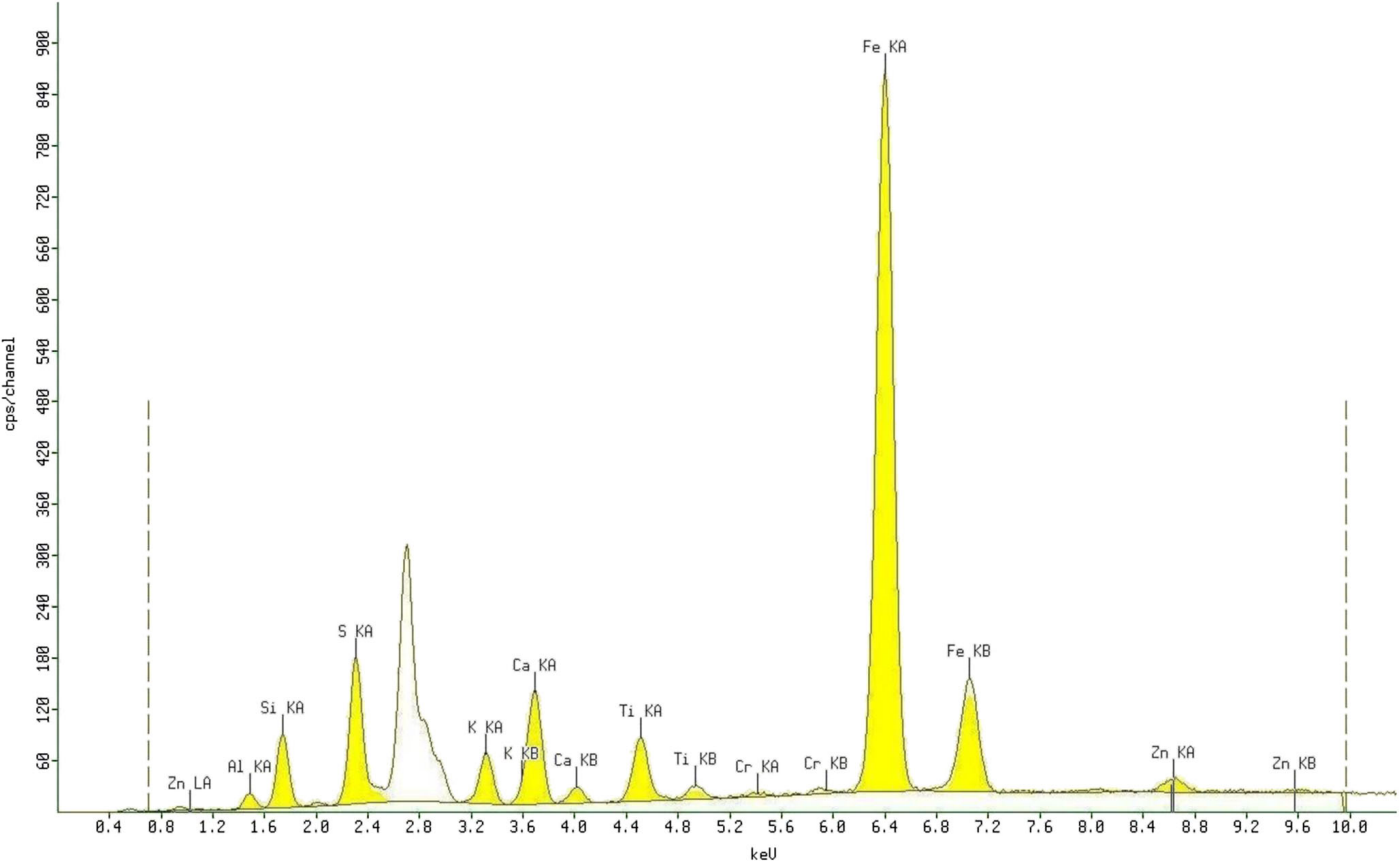
Exemplary XRF spectra of coal-water-glycerol suspensions (sample no. 24)

In all slurries made of coal material, the following elements were identified: Zn, Al, Si, S, K, Ca, Ti, Cr, and Fe (Fig. 3). These elements are from the raw material as well as from elements of the rotating mill.

Pareto charts were obtained. Based on them, it was concluded which input parameters (independent variables) or their interactions influence significantly (*α* = 5%) on output variables (viscosity, density, and carbon particle size in obtained suspensions).

Viscosity of suspension (for shear rate 100/min and 200/min) is significantly influenced by the multiple of glycerol and water content, water content (in cubic function) as well as time of grinding and multiple of type of raw material and grinding time. Raw materials are characterized by varying hardness. The harder the material, the harder it is to grind. Elongation of milling time causes that the milled material is more subjected to the grinding disks, which multiplies the effect of this process and the resulting product is characterized by a smaller particle size and resulting higher viscosity.

**Fig. 4:**
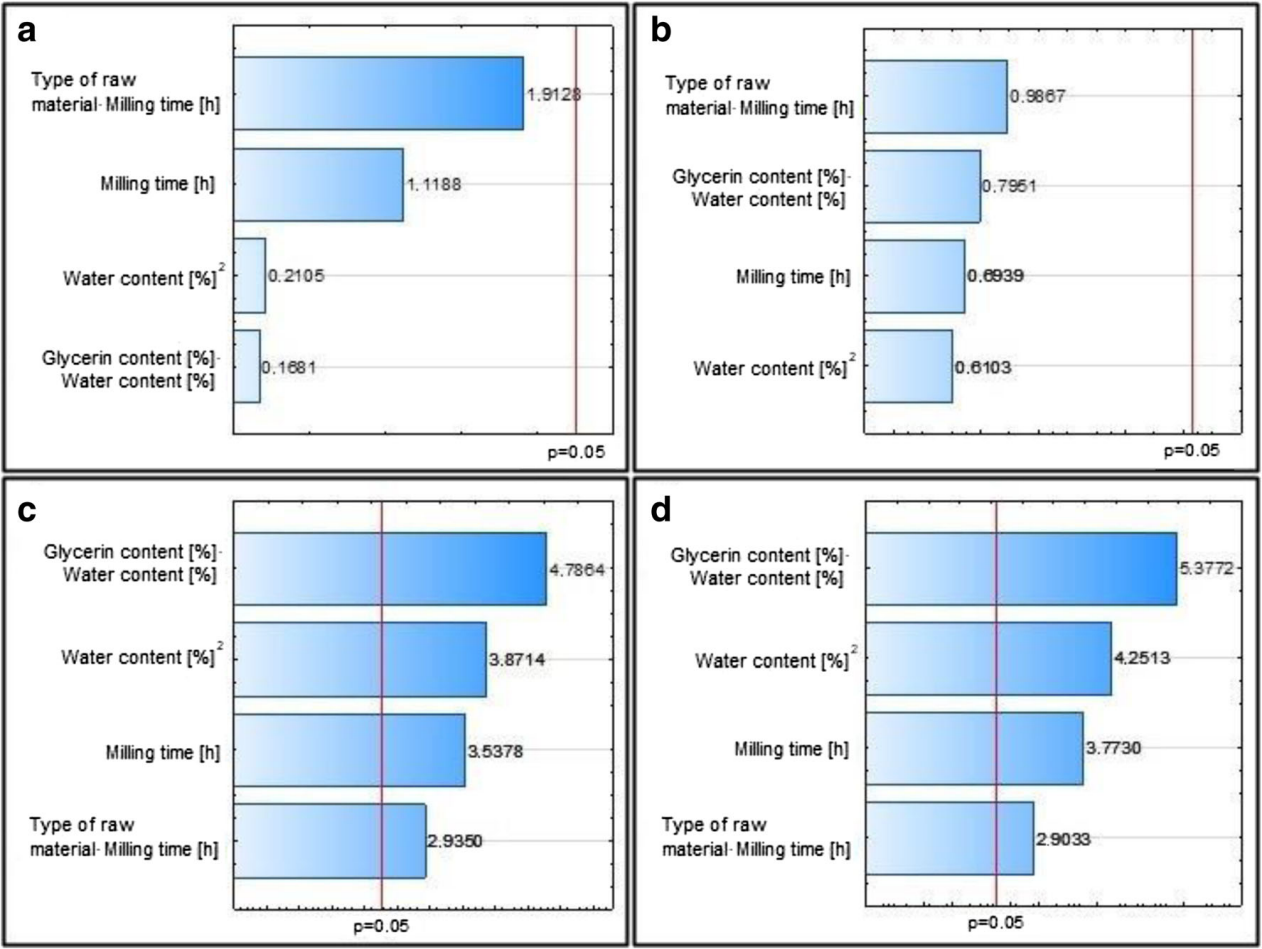
Pareto graphs of standardized results for the variables. **a** Substitution diameter. **b** Density. **c** Viscosity 100 rpm. **d** Viscosity 200 rpm

The water content in the slurry affects its viscosity (the lower the water content, the higher the viscosity). None of the independent variables has a significant effect on the coal particle’s diameter and the density of the obtained slurries (Fig. 4a–d).

**Fig. 5:**
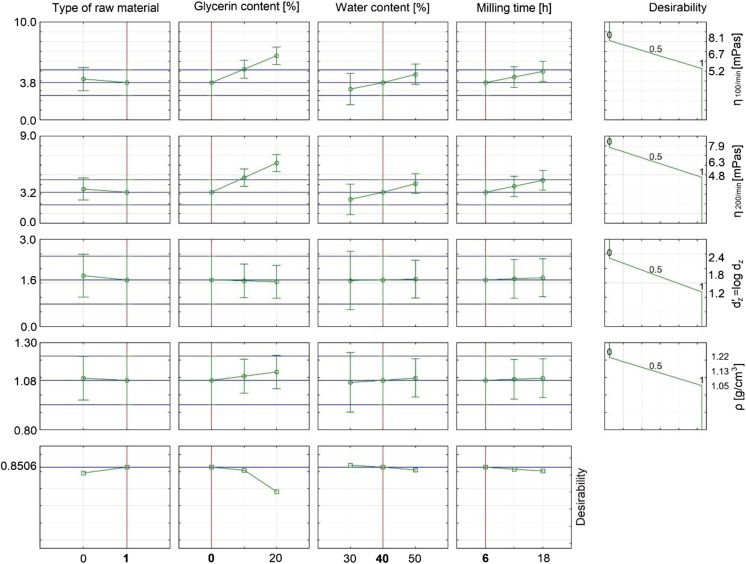
The approximation profile with the desirability function of the preparation process of coal-water slurry

The approximation profile and the utility function are shown in Fig. 5. The most advantageous values of the process parameters in the case of using coal as a coal raw material are the addition of 40% water, lack of glycerol, and 6 h of grinding. For these parameters, the viscosity of the resulting suspension is 45.5 mPa·s (for a shear rate of 100 rpm). At a shear rate of 200 rpm, the viscosity is 24.2 mPa·s and the equivalent particle diameter of the coal in the slurry is 5.0 μm and the density is 1.08 g/cm^3^. Desirability is about 0.85.

In order to increase the calorific value of the coal-water slurry, a portion of water was replaced by glycerol and the approximation function and utility function were determined. The most favorable values of the process parameters are 30% of water, 20% of glycerol, and 6-h grinding. For these parameters, the viscosity of the resulting suspension is 184.2 mPa·s for a shear rate of 100 rpm and 115.6 mPa·s for a shear rate of 200 rpm. The equivalent particle diameter of the coal particles is 4.6 μm and the density is 1.11 g/cm^3^. Desirability is 0.82.

By comparing the parameters of coal-water slurries and coal-water-glycerol slurries, it can be seen that the two-component system is characterized by more favorable viscosity values. Still, it is reasonable to obtain a coal-water-glycerol slurry because a slight decrease in useable properties is compensated by an increased calorific value of the product.

The most advantageous parameters of process for preparation of fine coal-water slurry were also determined. They are 40% of water, no glycerol added, and 6 h of grinding. For these parameters, the viscosity of the obtained suspension at a shear rate of 100 rpm is 65.1 mPa·s, a shear rate of 200 rpm gives 34.4 mPa·s, equivalent coal particle diameter is equal to 5.8 μm, and the density is 1.10 g/cm^3^. Usability is 0.78.

In order to increase the calorific value of the slurry, some part of water was replaced with glycerol and its impact on the useable properties of the resulting suspension was determined. The approximation function and the utility function for the fine coal-water-glycerol suspensions were determined. The most favorable values of the parameters for suspensions with fine coal were 30% of water and 20% of glycerol and 6 h of grinding. For these parameters, the viscosity of the resulting slurry at a shear rate of 100 rpm is 262.5 mPa·s, at a shear rate of 200 rpm of 163.9 mPa·s, the equivalent coal particle diameter is 5.4 μm, and the density is equal to 1.12 g/cm^3^. Usability is 0.71.

Comparing the parameters of fine coal-water suspensions and coal-water-glycerol suspensions, the lower viscosity and the lower density in a two-component suspension were observed. Similar data was observed by another researchers. They used glycerol to obtain slurry fuels from pine sawdust biochar. Liu et al. have investigated biochar-glycerol-water slurry fuels and they have observed that additive of glycerol leads to increasing of viscosity of these fuels (Liu et al. 2016).

The diameter of the coal particles in the dual and ternary suspensions is comparable. The addition of glycerol, which exhibits hygroscopic properties to the coal-water slurry, results in less moisture loss of these mixtures by evaporation. The higher viscosity of slurries with glycerol is due to the high viscosity index of glycerol (1410 mPa·s at 20 °C) relative to the water viscosity index (1.005 mPa·s at 20 °C). Glycerol solution at a concentration of 20% wt has a viscosity of 1.76 mPa·s at 20 °C. The same solution at 70 °C has a viscosity coefficient of 0.5 mPa·s (A glycerol 1990). Due to the fact that coal-water suspensions are dispensed into the heated furnace chamber, reducing the viscosity coefficient of water-glycerol solutions at higher temperatures will facilitate the nebulization of these suspensions. The increase in density of coal-water-glycerol slurries relative to the bicomponent slurry density results in a high density of glycerol compared to water density (sequentially 1.263 and 0.998 kg/m^3^). Obtaining of coal-water-glycerol mixtures is justified by the possibility of increasing the calorific value of the suspension. In addition, a 20% addition of glycerol in the coal-water mixture will reduce the freezing point of the slurry from 0 to − 5 °C (A glycerol 1990), which is extremely advantageous for transporting and storing it.

### Analysis of exhaust gas

**Fig. 6:**
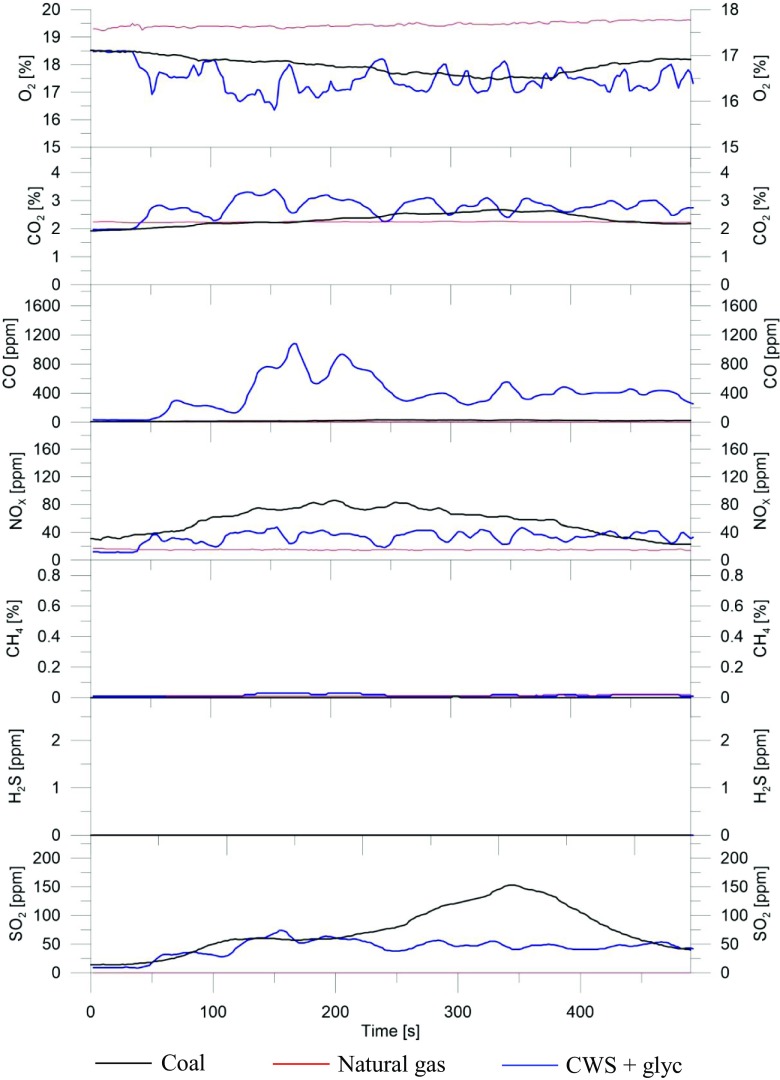
Analysis of the exhaust gases of the coal, natural gas, and coal-water-glycerol slurry combustion process (50% coal, 20% glycerol, 30% water obtained by 18-h grinding). Black line indicates coal, red line indicates natural gas, and blue line indicates CWS + glyc

Results of emission analysis changes in O_2_, CO_2_, CO, NO_*x*_, CH_4_, H_2_S, and SO_2_ in exhaust gases are presented in the graphs (Fig. 6).

The gas emissions while furnace is heated with natural gas and by coal or slurry combustion are presented in Fig. 6. In the case of natural gas, the content of the individual components in the flue gases is stable and is approximately equal to 18% of O_2_, 2% of CO_2_, and 30 ppm of NO_*x*_. No CO, CH_4_, H_2_S, and SO_2_ are observed. During combustion of coal in the analyzed gases, there are 17.5–18.5% of O_2_, 2–2.5% of CO_2_, up to 36 ppm of CO, up to 150 ppm of SO_2_, and up to 90 ppm of NO_*x*_. Gases emitted by the combustion process of the slurry, obtained by 18-h grinding of coal (50%) with water (30%) and glycerol (20%), are characterized by the following composition: 16.3–18.5% of O_2_, about 2–3.5% of CO_2_, up to 1100 ppm of CO, up to 75 ppm of SO_2_, up to 45 ppm of NO_*x*_, and up to 0.025% of CH_4_. Compared with conventional coal combustion, the content of nitric oxide and sulfur dioxide is lower than 50%. No H_2_S is observed. Similar studies were conducted by Dmitrienko et al. They compared the composition of the exhaust gases while burning fuel oil and coal-water slurries containing petrochemicals. Using the turbine oil as an additive for coal-based mixtures, they reduced the emissions of SO_*x*_ by 75% compared to the heating oil (Dmitrienko et al. 2018).

Based on the results of the emission analysis, it can be assumed that the coal-water suspension incineration resulting from coal micronization allows for a threefold reduction in SO_2_ emissions and a twice reduction in NO_*x*_ emissions. Emissions of remaining gases are comparable. The coal raw material of the slurry consists of potassium and calcium compounds that can contribute to the binding of sulfur compounds and the formation of a solid phase in the form of sulfates (IV). This reduces the emission of sulfur dioxide. According to Murko et al., it is possible to increase the efficiency of reduction of sulfur oxides in the flue gases during the combustion of coal-water suspensions by adding sulfur capture agents, whose action regards bonding of either sulfuric substances or compounds containing sulfur and formation new sulfuric compounds in the solid phase (Murko et al. 2016).

In the case of nitrogen oxides, they may be absorbed in water. Water-soluble nitrates are formed in liquefied water from gases. The high content of carbon monoxide in the flue gases determined during the research is due to the short residence time of the slurry particles in the combustion chamber. This is due to the method of dispensing the suspension fuel. This results in incomplete combustion of coal particles. This problem only affects laboratory conditions. And it can be solved in the case of changes in the type of combustion chamber or by increasing it. Increasing the combustion chamber will result in elongation of the residence time of the coal particles in the chamber at high temperature, which will allow oxidation of the carbon monoxide. Moreover, the addition of glycerol significantly reduced ignition delay time (Liu et al. 2016). Reducing the negative impact on the environment is one of the most important reasons for using coal-water suspensions as a substitute for coal. In addition, replacing coal with a coal-water mixture contributes to reducing ashes, thereby eliminating negative impacts on the respiratory system (Staroń et al. 2016).

## Conclusion

A method for obtaining stable suspensions containing coal, water, and glycerol was developed. The addition of glycerol to CWS increases the calorific value of the slurry and determines the useable properties of coal-water slurry and fine coal-water obtained in the disk mill, so that they can be a substitute for conventional fuels.

Coal-water slurry with glycerol is characterized by higher viscosity and density (184.2 mPa·s at a shear rate of 100 rpm, 1.11 g/cm^3^) than CWS without glycerol (45.5 mPa·s at a shear rate of 100 rpm, 1.08 g/cm^3^). Viscosity for fine coal-water slurry with glycerol was equal to 262.5 mPa·s at a shear rate of 100 rpm, and without glycerol was equal to 65.1 mPa·s at a shear rate of 100 rpm. Density values for these suspensions were respectively 1.12 and 1.10 g/cm^3^.

Emissions of nitrogen oxides and sulfur dioxide in coal-water slurry combustion are 50% and 60% lower than for coal. During coal-water-glycerol slurry incineration, the amount of oxides emitted relative to the amount of these gases emitted during coal combustion is reduced by half. Waste materials such as coal sludge or biomass can also be used to produce CWS without prior drying. This may be the basis for further research.
